# Feature Selection for Continuous within- and Cross-User EEG-Based Emotion Recognition

**DOI:** 10.3390/s22239282

**Published:** 2022-11-29

**Authors:** Nicole Bendrich, Pradeep Kumar, Erik Scheme

**Affiliations:** Institute of Biomedical Engineering, University of New Brunswick, Fredericton, NB E3B 5A3, Canada

**Keywords:** affective computing, EEG, emotion classification, AMIGOS dataset, machine learning, feature selection

## Abstract

The monitoring of emotional state is important in the prevention and management of mental health problems and is increasingly being used to support affective computing. As such, researchers are exploring various modalities from which emotion can be inferred, such as through facial images or via electroencephalography (EEG) signals. Current research commonly investigates the performance of machine-learning-based emotion recognition systems by exposing users to stimuli that are assumed to elicit a single unchanging emotional response. Moreover, in order to demonstrate better results, many models are tested in evaluation frameworks that do not reflect realistic real-world implementations. Consequently, in this paper, we explore the design of EEG-based emotion recognition systems using longer, variable stimuli using the publicly available AMIGOS dataset. Feature engineering and selection results are evaluated across four different cross-validation frameworks, including versions of leave-one-movie-out (testing with a known user, but a previously unseen movie), leave-one-person-out (testing with a known movie, but a previously unseen person), and leave-one-person-and-movie-out (testing on both a new user and new movie). Results of feature selection lead to a 13% absolute improvement over comparable previously reported studies, and demonstrate the importance of evaluation framework on the design and performance of EEG-based emotion recognition systems.

## 1. Introduction

According to the Mental Health Commission of Canada, 50% of Canadians have or will have had a mental health illness by the age of 40 [1], with mood and anxiety disorders among the most common types of mental health disorders [2]. Given that individuals with mental health illnesses respond well to early intervention [3], there is hope that early awareness could play a significant role in supporting the mental health needs of Canadians. Early detection of frequent changes in emotions has been recognized as a cornerstone in the treatment of both mood and anxiety disorders. To this end, researchers in the field of affective computing have been developing systems to elicit and recognize emotion using various modalities. For example, Mower et al. determined emotions from the audio and visual data of actors as they performed emotionally evocative scripts [4]. Valstar and Pantic showed short films to induce disgust, happiness, and surprise in participants [5], and used the Face Action Coding System (FACS) to determine the experienced emotions. Niu et al. used songs to induce emotions in participants while collecting physiological signals (electrocardiography (ECG), galvanic skin response (GSR), electromyography (EMG), respiration (RESP)) [6]. Likewise, He et al. collected physiological signals but induced emotions using video clips [7]. The authors noted the benefit of physiological signals (ECG, RESP) for individuals experiencing mental illnesses, as these individuals may avoid exhibiting changes in facial expressions, tone of voice, body posture, and gestures. Since emotion is a highly cognitive process, it is particularly valuable to understand how electroencephalography (EEG) signals relate to emotion. Several studies have used EEG for emotion recognition as its measurement uses relatively inexpensive, noninvasive technology that yields high temporal resolution [8,9,10]. Due to the increased demand for Human–Computer-Interaction (HCI) and the desire to understand emotions, the field of affective computing has been growing in recent years.

Despite this growth, it can be argued that the field remains limited in applicability due to the experimental conditions employed in most research. For example, the majority of emotion recognition studies are conducted using stimuli that last less than ten minutes [11,12]. Generally, studies are based on short stimuli, as psychologists have recommended eliciting discrete emotions to alleviate challenges with obtaining ground truth labels for the data [13,14]. The use of such short stimulus presentation to participants, however, cannot then reflect the temporal and contextual evolution and continuum of emotions. This is further confounded by the nature of label generation. For example, it is common practice in existing studies to have participants provide an annotation (i.e., happy or sad) at the end of the period of stimulation [15,16]. This is necessary because having them continuously record annotations throughout the stimulus would preclude immersion for the subject, thus changing the observed emotions. Providing retrospective annotations, however, is highly error-prone and so participants are commonly asked to provide only a single label for the entire period. This only further adds to the challenge of understanding the evolution of emotion over time. Although longer stimuli may provide a more dynamic range and context of emotion, potentially facilitating a better understanding of emotion, they are complicated by the above mentioned challenges associated with accurate labeling. The objective of this work is, therefore, to investigate how different design factors impact the performance of EEG-based emotion recognition systems in the context of longer stimuli.

Furthermore, in the literature, emotion recognition performance is typically evaluated using cross-validation schemes such as leave-one-person-out (LOPO), k-fold, or leave-one-sample-out (LOO) [17,18]. Cross-validation schemes like LOPO (which are applicable only when a user is exposed to a known, previously seen stimulus) provide limited insight into how well a laboratory-created model may generalize to the real-world. However, other validation schemes such as leave-one-movie-out (LOMO) and leave-one-person-and-movie-out (LOPMO) are considered better representations of the generalizability of a system [19]. Specifically, the LOPMO scheme has not yet been widely adopted in the literature, presumably because it severely reduces the observed performance of emotion recognition systems. Nevertheless, LOPMO best reflects the goal of a real-world implementation, as it would represent a truly generalizable subject- and stimulus-independent affective computing system. Consequently, in this work, we evaluate the performance of the developed systems in the context of each of these frameworks to evaluate the continuum between possible and probable performance.

Independent of framework, previous works have shown many useful features from the time, frequency, and time–frequency domains that carry effective information for recognizing different emotions from EEG. More recently, features such as higher-order crossings, higher-order spectra, etc. have been shown to outperform common features like power spectral bands [20]. No standard, agreed upon, subset of these features has been established for EEG-based emotion recognition, but the naive application of all features could lead to issues with dimensionality [21]. Consequently, different feature selection methods (filter & wrapper) have been shown to be effective in automatically selecting the best features given a set of design considerations. Although they can risk over-tuning, wrapper-based feature selection methods are often preferred as they iteratively assess interactions between features enabling them to select combinations of features that may seem irrelevant when considered individually in filter-based methods [21]. In this work, we implement two simple and widely used wrapper-based feature selection techniques; sequential forward selection (SFS) and sequential backward selection (SBS). Both of these techniques are effective at selecting salient features that reduce redundancy and improve the discrimination of the classification problem [22].

The main contributions of the paper are, therefore, as follows.

- An analysis of existing EEG features and their selection for recognizing time-varying emotions induced by longer stumuli.
- An evaluation of the impact of various cross-validation evaluation frameworks, and thereby use cases, on the design and performance of EEG-based emotion classification.

## 2. Related Work

EEG has been considered by many researchers as a prominent modality in the development of emotion recognition systems [23,24], in part due to the substantial role the brain plays in regulating and processing sensory inputs and emotion [25]. As with other modalities, however, the reliability of obtaining accurate ground-truth labels and the duration and intensity of elicited emotions, remain ongoing challenges in the pursuit of automatic affect recognition systems. For these reasons, most studies have limited experiments to short stimuli, during which emotions are assumed to be static or stationary. To facilitate this assumption, many research groups have used stimulus lengths of less than 10 min [11,12]. Conversely, in real life, people experience continuous and mixed emotions in response to dynamically varying stimuli, resulting in combinations of, and transitions between, emotions. This consideration motivates the exploration of the temporal dynamics of emotion in affective computing, but raises substantial challenges with labelling. Although some researchers have attempted continuous labelling, most of the resulting work has focused on computer vision applications, and not physiological signals such as EEG [26,27]. In 2016, Soleymani et al. [28] recorded EEG signals from subjects watching short movies, and external annotators were subsequently asked to label frontal-view videos of the participants. More recently, the AMIGOS [29] dataset was released, comprising ECG, EEG, and GSR data from both short movies (less than 150 s) and longer movies (greater than 14 min). The dataset includes a time series of emotion labels for the long movies as determined by three external annotators at twenty-second intervals.

Various machine learning techniques have been investigated to improve the performance of emotion recognition and other health related systems (e.g., stroke management) using EEG [30,31]. Many classical classifiers have been proposed, including support vector machine (SVM) [32,33,34], linear discriminant analysis (LDA) [33,34], k-nearest neighbours (kNN) [33], random forest (RF) [35,36], Naïve Bayes (NB) [32,36], extreme gradient boosting (XGB) [32], and decision trees [33,36]. The performances achieved by these classifiers vary, and differences are often overshadowed by the impact of the features chosen as part of these models. In recent deep learning works, convolutional neural networks (CNN) [18,34,37], recurrent neural networks (RNN) [18], and extreme learning machines (ELM) [24] have all been employed with varying degrees of success. For example, in [38], a multimodal learning framework was developed using EEG-based spectral topographic maps and facial-expression based action units. The authors showed that facial expressions have a strong correlation with the emotionally significant EEG features across multiple datasets. Likewise, inter-channel relationship between EEG signals was recently modeled in [39] using regularized graph neural networks, using adversarial training and emotion-aware distribution learning to handle cross-subject EEG variations and noisy labels, respectively.

Whether engineered, such as with handcrafted features, or learned, such as with deep learning, the extraction of features is a critical step in increasing the information density of the signal prior to classification. Most commonly, handcrafted features are extracted from specific frequency bands in the signal (e.g., the alpha, beta, delta, gamma, and theta bands are used in 89.4% of works) [12]. To extract these features, frequency (Freq) domain techniques such as power spectral density (PSD) [40,41] and asymmetry (Asymm) [20] between electrodes have been proposed. Time-domain features can also be extracted, such as high order crossings [42] and Hjorth parameters [35,43]. Non-linear methods can be used to extract various entropy measures [44] and fractal dimensions [44,45], and more recently, features have been learned automatically using deep learning approaches [24]. Jenke et al. [20] reviewed various features extracted from EEG signals, and determined that complexity measures such as fractal dimensions are important for emotion recognition, but suggested that further investigation is needed to better understand the impact of different groups of features. Similarly, despite their potential benefits, features learned using deep learning model currently lack interpretability [46].

Despite the known body of features, there is little consensus on a defined set of the most appropriate features for EEG-based emotion recognition. Very few works exist that either compare different features [42] or apply some form of feature selection technique [47]. In machine learning, the selection of features with high information density is essential to improve performance and avoid the ‘curse of dimensionality’, wherein the training data become sparse due to the high dimensionality introduced by the large number of features. Atkinson et al. applied the statistical minimum-Redundancy-Maximum-Relevance (mRMR) approach to eliminate redundant features [48]. Similarly, one-way ANOVA [36] and other classical filter based approaches were applied in [43] to select optimal features. Other techniques explored include evolutionary computation [21], transfer recursive feature elimination (T-RFE) [49] using geometrical distances and locally-robust feature selection (LRFS) using probability densities of extracted features. Jenke et al. [20] performed a comparison between different feature and EEG electrode selection techniques on a self-recorded dataset. In each of these works, however, they either extracted very few features or used relatively small datasets. Furthermore, these approaches were all applied to short stimuli, under the assumption that they emotion was static.

When evaluating these systems, many of the existing works have adopted k-fold or leave-one-sample-out (LOO) techniques [11]. In these approaches, parts of the same data sequence, sometimes even consecutive frames, may be selected for both training and testing, resulting in an emotion recognition performance that is dependent on having previously seen a specific stimulus. Consequently, two other approaches are also commonly adopted. The first method, the participant agnostic (independent) approach, is used in the largest proportion of the literature [32,50]. Because it is designed to extend to previously unseen participants (LOPO), the generalizability of this approach could make it more tangible for real-world adoption. Such participant-independent systems, however, have often obtained F1-scores (harmonic mean of precision and recall) between 0.5 and 0.6 [50,51,52], leaving considerable room for improvement. Furthermore, although the testing subject may be previously unknown in this evaluation framework, the data for all subjects are commonly collected using the same stimuli. Consequently, this effectively limits the generalizability of the results to new people only while they are experiencing a known stimuli that is consistent with that used to train the model.

The second method focuses on developing a model that is tailored for a given participant (participant-dependent or within-subject). The participant-dependent approach has yielded substantially better performance [11,53], typical F1-scores fall between 0.6 and 0.8 [54,55], likely due to the avoidance of inter-subject variability. Although within-subject models produce better results, their real-world applicability may be limited because they require a model to be trained specifically for each user. Although this could be viable for select high-impact applications, the necessity to collect person-specific labeled training data precludes most commercial applications. Consequently, although both of these common validation frameworks provide some insights about emotion recognition, further consideration of the problem is needed to increase the generalizability of the results and their application.

## 3. Methods

### 3.1. Dataset & Preprocessing

**Figure 1:**
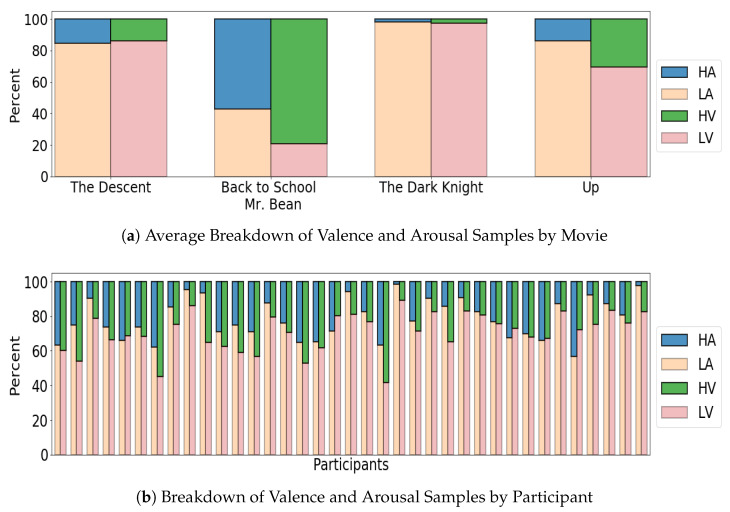
Class Breakdown on a Per Movie and Per Participant Basis.

In this work, a publicly available dataset AMIGOS [29] was used to evaluate EEG-based emotion recognition performance across a range of validation frameworks. The AMIGOS dataset was recorded using the Emotiv EPOC headset, comprised of 14 channels according to the international 10–20 system at positions AF3, F7, F3, FC5, T7, P7, O1, O2, P8, T8, FC6, F4, F8, AF4. The dataset was collected from 40 participants, recorded while they watched 16 different short films (<150 s each) and excerpts from 4 longer films (>14 min each). The short videos were watched individually in isolation, whereas some participants watched the long films together in groups. In this work, we focused only on the EEG data corresponding to long movies as it contained time-varying labels and is arguably more representative of a real-world scenario. The details of the long movies used to record EEG data is presented in Table 1 along with their duration. The division of high/low valence and arousal categories was achieved using the zero affect threshold as proposed in the original AMIGOS work [29] (Figure 1a shows the percentage of samples annotated as high/low valence and arousal). It may be noted that Mr. Bean had the most high affect samples, with around 79% high valence samples and around 57% high arousal samples. The Dark Knight had the most low affect samples, with 97% low valence samples and 98% low arousal samples. The participant-specific breakdown of these results is shown in Figure 1b. It can be seen that eight participants had less than 10% high arousal, and one participant (#23) was assigned less than 1%. Valence was found to be slightly more balanced than arousal but still tended toward low valence samples.

The AMIGOS signals were previously preprocessed using average-referencing and high-pass filtering with a 2 Hz cut-off frequency. Motion artifacts from eye movements were also previously removed using a blind source separation technique [56].

The ground truth labels were generated by three external annotators by examining the facial expressions of the participants as they watch the movies. These external labels were created by each annotator for every non-overlapping twenty-second period. The facial expressions were rated by the annotators using the continuous valence and arousal scales, according to Russell’s Circumplex Model of Affect [57]. When considering the assigned binary class labels, the annotators were found to agree 75% of the time for valence and 61% of the time for arousal. Furthermore, the majority of samples that were agreed upon fell into the low affect classes, leaving heavy disagreement in the high affect cases. Briefly, valence quantifies the range of positive and negative emotion, which ranges from pleasant to unpleasant, whereas arousal quantifies the level of engagement from passive to active, indicating the intensity of affect. Participants 8, 24, and 28 were missing the external annotations for all movies and were, therefore, not used in this work. The first and last labels for each movie were discarded to ensure non-overlapping labels leaving a set of 14 different time-series of twenty-second windows of EEG (one for each channel) from which features were extracted.

### 3.2. Feature Extraction & Selection

In addition to the power spectral density (PSD) and differential spectral power asymmetries features (105) extracted by Miranda et al. as part of their original work [29], another 112 features were identified from the literature and compiled from various sources. Briefly, these included the *fractal dimension* (2 methods), *entropy* (2 methods), *Hjorth parameters* (2 methods), *detrended fluctuation analysis*, and *Fisher information*. Thus, a total of 217 features (combinations of feature methods and channels) were analysed in this work. Next, feature selection techniques were applied to remove the redundant informative, increase information density, and thus, reduce the risk of overfitting. The details of the feature extraction selection are described as follows.

#### 3.2.1. Feature Extraction

- **PSD**: The power spectral density, reflective of the distribution of signal power across frequencies [58], is one of the most common EEG features. The PSD was calculated for different EEG frequency bands, as different bands have been associated with different processes [12]. Theta waves are associated with affective processing [59], whereas the slower alpha band reflects attentional demands such as alertness and expectancy [60]. The entire alpha band reflects task-related processes [60]. Alpha waves tend to occur when someone is in a relaxed state of mind, while beta waves tend to occur when an individual is in more of an active state [61]. The gamma band reflects a reflective aspect when processing emotional material [62].
  Following AMIGOS [29], the Welch method with windows of 128 samples (1 s) were used to calculate the PSDs. These PSDs, *X*, were then averaged over each frequency band, and the logarithms were obtained as features. The terms ‘low’ and ‘high’ refer to the minimum and maximum frequency range within a band.
  (1) $${\mathrm{PSD}}_{f_{\mathrm{low}},f_{\mathrm{high}}}=\log\left(\frac{1}{f_{\mathrm{high}}-f_{\mathrm{low}}}\sum_{f=f_{\mathrm{low}}}^{f_{\mathrm{high}}}X\left[f\right]\right),$$
- **Spectral Asymmetry (Asymm)**: Spectral asymmetry leverages both frequency-domain and spatial information about emotional changes in the brain [41]. The asymmetry in the different bands across channels has been shown to be indicative of different emotions. For example, the alpha asymmetry between frontal lobe channels F3 and F4 can relate to valence [63] and the beta asymmetry between parietal lobe channels P3 and P4 can correlate with angry facial expressions [64].
  The differential spectral asymmetry was calculated by taking the difference of the PSD features from symmetric channels from the left and right hemispheres
  (2) $${\mathrm{Asymmetry}}_{f_{\mathrm{low}},f_{\mathrm{high}}}^{a,b}={\mathrm{PSD}}_{f_{\mathrm{low}},f_{\mathrm{high}}}^{a}-{\mathrm{PSD}}_{f_{\mathrm{low}},f_{\mathrm{high}}}^{b},$$
  where *a* and *b* represent different EEG channels. For example, a slow alpha asymmetry for parietal lobe channel P3 and P4 is ${\mathrm{Asymmetry}}_{8,10}^{P3,P4}$.
- **Hjorth**: The Hjorth Mobility (HM) is an estimation of the signal’s mean frequency, and the Hjorth Complexity (HC) reflects the bandwidth and the change in frequency [65]. It is defined as the square root of the variance of the signal derivative, normalized by the variance of the signal, while not yet widely adopted, Hjorth features have been shown to be relevant for emotion recognition [43]. Equations are as given in [66].
- **Detrended Fluctuation Analysis (DFA)**: DFA quantifies the statistical persistence, or auto-correlation, property of non-stationary physiological signals [67]. Briefly, DFA evaluates the detrended and integrated signal as a function of window size. Commonly used in many fields, including for ECG analysis, DFA has also been found to be beneficial for EEG emotion recognition [68]. Equations are as given in [66].
- **Fractal Dimension (FD)**: Fractal dimension approaches, such as the Petrosian fractal dimension (PFD) and Higuchi fractal dimension (HFD), are a measure of signal complexity [69] and are commonly used for non-stationary and transient signals. The Higuchi fractal dimension has been used more frequently in emotion recognition works [12], but in neurophysiology, both Higuchi and Petrosian fractal dimensions are commonly cited [70]. Equations are as given in [66].
- **Entropy (Ent)**: Entropy is a measure of chaos, or disorder, in a system or signal, and is, therefore, used to understand signal complexity [71]. Here, the spectral entropy (SpecEnt), the entropy of the PSD [72], and the SVD entropy (svdEnt), an indicator of how many vectors are needed to reconstruct an adequate explanation of a signal [72], were used. Hatamikia et al. [44] found that spectral entropy outperformed the Petrosian and Katz fractal dimensions for emotion recognition. Gupta et al. [35] used SVD entropy as part of a set of features to classify discrete emotion for short movies. Equations are as given in [72].
- **Fisher Information (FI)**: The Fisher Information is a measure of how much information a random variable carries about the data that it models. It is also known as the expected value of the observed information [73]. Although less commonly used, FI of EEG has been shown to contain affect information [35]. Equations are as given in [66].

#### 3.2.2. Feature Selection

To identify which features were most relevant and informative for emotion recognition, two common greedy-search feature selection techniques, namely Sequential Forward Selection (SFS) [74] and Sequential Backward Selection (SBS) [75], were implemented. The SFS method starts with an empty set of features and incrementally adds a feature to the set. The feature that is added at each step is the one that gives the largest performance improvement when added to the existing set. Conversely, SBS begins with the group of all features and iteratively removes one feature at a time. The feature removed at each step is the one that leaves the highest performance for the remaining set. It should be noted that, although faster than an exhaustive brute-force search, both SFS and SBS (due to their wrapper-style objective functions) may miss elements of information in the relationships between groups of features. Nevertheless, they are both commonly used and, when interpreted carefully, can lead to important understanding and performance benefits. In this work, feature selection was conducted using SFS and SBS, beginning from the set of 217 features, and the best set for each case was determined as the set that achieved the maximum classification performance.

### 3.3. Emotion Classification & Evaluation Metrics

Virtually all classifiers have been used in some form in emotion recognition [11,12]. The most common classifier, however, is the Support Vector Machine (SVM), reported to have been used in 59% of cases by a recent review [12]. Because of its familiarity in the field and its inherent trade-off between accuracy and generalization [40], SVM was adopted in this work using a linear kernel. Although less frequently used (6.3% of emotion research [12]), Linear Discriminant Analysis (LDA) is also robust and less computationally intensive. LDA models the distributions of the class data, whereas the SVM classifier focuses explicitly on the data at the boundaries between classes.

For comparison, a deep learning-based scheme was also evaluated. Because EEG data is spatio-temporal in nature, a convolutional long short term memory (CLSTM) architecture was employed using 1D CNNs and LSTMs [76]. 1D CNN layers were used to encode spatial representations of the EEG signal, which were then subsequently processed temporally using LSTM layers. The designed network consisted of two CNN layers with 64 and 128 filters of sizes 10 and 5, followed by 2 max-pooling layers with size 3. The learned feature embeddings were then passed to the stacked LSTM layer, comprised of 256 units followed by three dense layers with 256, 128, and 1 neurons. The final emotion classification was completed using a final dense layer governed by a Sigmoid activation. The network was trained with a binary cross-entropy loss function with an initial learning rate of $1\times 10^{-3}$.

Using the LDA, SVM, and CLSTM classifiers, emotion recognition was accomplished as a set of two 2-class problems; high vs. low valence and high vs. low arousal. That is, valence and arousal models were trained independently of each other using binarized versions of the continuous valence and arousal labels provided with AMIGOS. Negative values of valence and arousal were assigned to corresponding the “low” class, whereas positive values were assigned to the corresponding “high” class.

The conventional classification accuracy performance metric is prone to bias when class distributions are unbalanced (as is the case with the AMIGOS dataset). Consequently, in this work, F1-score was used to describe the classification performance of the various models. The F1-score is described as the harmonic mean of precision and recall as defined in Equation (3). Precision, as shown in Equation (4), determines how many instances of the positive predictions came from the positive class, making it a measure of exactness. Recall, as shown in Equation (5), determines how many instances of the positive class are successfully predicted as positive, making it a measure of completeness.

(3) $$F1-\mathrm{score}=2\;\frac{\mathrm{Precision}\cdot \mathrm{Recall}}{\mathrm{Precision}+\mathrm{Recall}}$$

(4) $$\mathrm{Precision}=\frac{TP}{TP+FP}$$

(5) $$\mathrm{Recall}\;\left(\mathrm{Sensitivity}\right)=\frac{TP}{TP+FN}$$

TP: True Positive, TN: True Negative, FP: False Positive, FN: False Negative.

### 3.4. Evaluation Frameworks

While k-fold and LOO approaches are popular in the literature, it has been suggested that for participant-independent results, frameworks such as Leave-One-Person-Out (LOPO) may be more appropriate [77]. In this framework, the system is trained with all subjects but one and tested with the previously unseen subject. This process is repeated until all subjects have been tested, and the results are averaged across all cases.

Within the participant-dependent approach, the k-fold approach has been more prevalent [11] although some works have also used LOO [55,78]. Depending on their application, however, these approaches may also provide the classifiers with training information about a known stimulus, as previously described. Consequently, in this work, any discussion of participant-dependent results are based on a stimulus-independent Leave-One-Movie-Out (LOMO) framework, meaning that all movies but one are used for training, and testing is performed on the remaining, previously unseen, movie. This process is repeated until all movies have been tested, and the results are averaged across all cases, while not as popular as LOPO, the LOMO framework has recently been explored in the literature. Malandrakis et al. [26] and Baveye et al. [27] used LOMO with audio and video features to classify affective states. Similarly, Tian et al. [79] used the LOMO scheme to investigate how well audio, video, and GSR features can determine the level of induced valence and arousal in an audience.

One important consideration with the LOPO approach is that it is most often applied for a given stimulus. For example, although the testing subject may be previously unknown, the data for all subjects are collected while they watch the same movie. Again, this conceptually limits the generalizability of the results to new people, but only during known stimuli. A truly subject and stimulus-independent Leave-One-Person-And-Movie-Out (LOPMO) scheme has not yet been widely adopted in the literature, presumably because it severely reduces the observed performance of emotion recognition systems. Nevertheless, LOPMO best reflects the goal of a real-world implementation, as it would represent a truly generalizable subject- and stimulus-independent affective computing system. Indeed, even in 2011, Kolodyazhniy et al. [19] suggested the need for a subject and stimulus-independent classification. In their work, they used ten-minute movies that elicited fear, sadness, and neutral emotional states, and a total of 14 features derived from ECG, GSR, respiration, temperature, and EMG. Their work, however, did not employ EEG or use continuous labels.

In summary, these evaluation frameworks can be categorized as shown in Table 2. Note that the real-world applicability of these frameworks increases from a minimum in the top left (known subject and stimulus), to a maximum in the bottom right (LOPMO). It is important to note the differences in the amount of training and testing data available for the different validation schemes, as presented in Table 3. In this paper, results are presented for the LOPO, LOMO, and LOPMO cross-validation evaluation frameworks.

### 3.5. Statistical Testing

For each cross-validation framework, results were tested for normality using the Kolmogorov–Smirnov test [80]. Because each framework was found to be non-normal, significance of results was subsequently evaluated using the non-parametric Kruskal–Wallis H-test [81].

## 4. Results

As outlined above, the performance of the combinations of feature selection techniques (all features, SFS, or SBS) and classifier (LDA or SVM) were evaluated for each of the different cross-validation frameworks (LOPO, LOMO, or LOPMO). The results of these analyses are then compared with CLSTM-based classification as described as follows.

### 4.1. LOPO Cross-Validation

To evaluate the LOPO scheme, a new model was trained for each fold using all participants but one, and tested using that remaining participant. This process was repeated until each participant had been tested and the average result across all folds was recorded. Table 4 shows the results of these analyses when using SFS, SBS, or the full feature set. Note that the corresponding number of features chosen during feature selection is also shown for each configuration (with 217 being all features).

**Figure 2:**
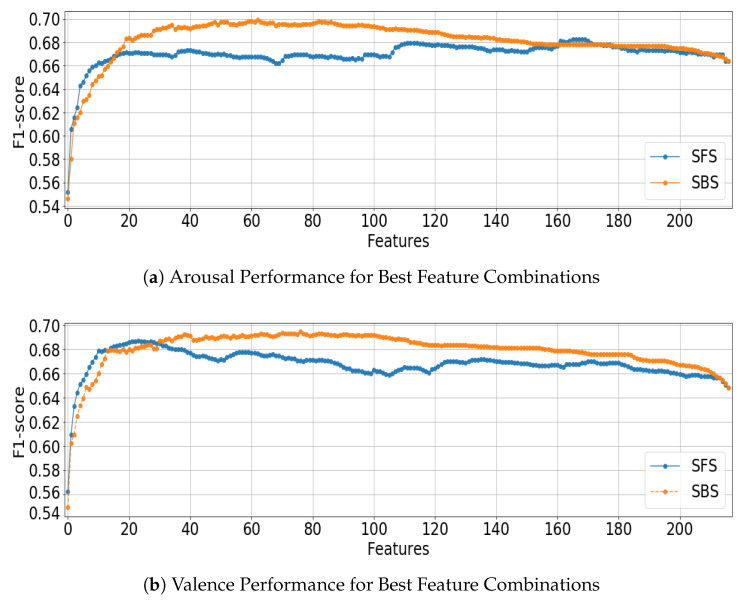
Comparison of SFS and SBS Approaches for Classification of Valence and Arousal Using LOPO LDA.

Using the Kruskal–Wallis H-test, no significant difference was found between the results of SFS and SBS when using an LDA or SVM classifier with the chosen number of features ([LDA, SVM]: arousal *p* = [0.585, 0.570], valence *p* = [0.931, 0.277]), however, Figure 2 shows the different selection profiles of the approaches. Because SFS cannot predict correlations between features, it can be seen to be less ‘smooth’ in its performance improvements. Conversely, SBS starts with all features and removes the worst-performing feature, helping it to better understand feature relationships. These results suggest that there is meaningful information in the correlation between channels and features that plays an important role in EEG-based emotion recognition. The CLSTM network achieved comparable performance, yielding a valence F1-score of 0.035 more than the SVM using SBS classification result, and 0.022 less in arousal F1-score than the LDA using SBS. Using the Kruskal–Wallis H-test, the CLSTM results were compared against the CLF, ALL results. A significant difference was found for valence, but not for arousal ([LDA, SVM]: arousal *p* = [0.717, 0.245], valence *p* = [0.002, 0.000]).

The top five features selected for LOPO cross-validation via SFS and SBS are presented in Table 5. Features are named according to the following naming convention: [Electrode, Feature, Band]. For asymmetry features, the lobe is specified as opposed to the electrode. Only PSD and Asymm features have specified bands. Interestingly, although some of the features originally proposed by [29] were selected, the majority (and first) of those chosen were previously unused with the AMIGOS dataset. The bottom row of each column shows the F1-score when using these five features, and the percentage of the maximum score these five features achieved. In each case, using only five features achieved over 89% of the best F1-score for each model. For many of the models, HFD, the Higuchi Fractal Dimension, was found to be the most important feature. This corroborates the findings of previous works, supporting that HFD is an important feature for EEG-based emotion recognition [45,82].

### 4.2. Lomo Cross-Validation

Within the LOMO framework, two different schemes were evaluated; LOMO-Inter and LOMO-Within. The term LOMO-Inter is used to refer to the inter-participant LOMO case, wherein a model is trained using multiple users, but evaluated on a previously unseen stimulus (in this case, movie). The term LOMO-Within is used to refer to the within-participant LOMO case, which is similar, but wherein a unique model is trained for each participant.

#### 4.2.1. LOMO-Inter Cross-Validation

The LOMO-Inter cross-validation classification results, along with the number of features selected using SFS and SBS, are shown in Table 6. These results reflect the average of 4 folds, with each fold trained using three movies from all 37 people (8, 24, 28 excluded, as in LOPO) and tested using the remaining movie.

For both LDA and SVM, SBS trended towards outperforming SFS, but not significantly ([LDA, SVM]: arousal *p* = [0.564, 0.564], valence *p* = [0.773, 0.773]). Similarly, no significant differences were found between LDA and SVM ([SFS, SBS, ALL]: arousal *p* = [0.564, 0.564, 0.564], valence *p* = [0.773, 1.000, 0.773]). Table 7 shows the top five features selected by SFS and SBS for the LOMO-Inter framework. From these tables, it is again evident that the new features extracted in this work play a significant role in the classification performance. Using five features, over 87% of the maximum F1-score was achieved in all cases. As was observed for LOPO, the fractal dimension features were again highlighted as important for the emotion recognition problem. For CLSTM, F1-scores of 0.612 and 0.603 were recorded for valence and arousal classification, respectively. Table 6 shows that the SVM and LDA classifiers outperform CLSTM in both cases. The limited training data in the cross-validation scheme may have affected the CLSTM as data for only three movies were available to train the classifier. Using the Kruskal–Wallis H-test, the CLSTM results were compared against the CLF, ALL results. No significant differences were found ([LDA, SVM]: arousal *p* = [0.564, 0.564], valence *p* = [0.564, 0.564]).

#### 4.2.2. Lomo-Within Cross-Validation

The LOMO-Within cross-validation results, wherein each model was built using a single participant, are shown in Table 8 along with the number of features selected using SFS and SBS. Again participants 8, 24, and 28 were excluded as they did not participate in the long movie experiments. The use of a single participant also caused additional problems. Participants 17, 18, and 22 were missing annotations for some long movies, and were consequently excluded. Similarly, due to extreme imbalances in the ratio of the high and low classes (less than 10 high affect samples, representing approximately 5.5 to 6.5% of decisions), participants [20, 21, 23, 26, 31, and 38–40] were also excluded for valence and [11–13, 15, 20, 21, 23, 25–27, 29, 30, 35, 37, and 40] were excluded for arousal. LOMO-Within models were trained for each participant using data from three movies and tested on the same participant’s data for the fourth movie in a leave-one-out framework. Results represent the average across all four movies and across 26 participants for valence and 19 participants for arousal.

For both the SVM and LDA models, the feature selection techniques significantly improved the classification performance over the all features case (*p* < 0.01). This reinforces the importance of feature selection to overcome the curse of dimensionality, particularly in cases where there is with comparably little data. The top five features selected by SFS and SBS for the LOMO-Within validation scheme are presented in Table 9. The limited training data is also reflected in the performance of CLSTM classifier, where F1-scores of 0.543 and 0.579 are recorded for valence and arousal classification, respectively. However, CLSTM still outperformed the baseline cases, when all features were employed without feature selection. Using the Kruskal–Wallis H-test, the CLSTM results were compared against the CLF, ALL results. Significant differences were found for both valence and arousal ([LDA, SVM]: arousal *p* = [0.000, 0.037], valence *p* = [0.000, 0.010]).

### 4.3. Lopmo Cross-Validation

The LOPMO cross-validation framework evaluates how well a model may generalize to both a new person and a new stimulus. This makes it the most challenging, but also the most representative of potential real-world performance. The results using the LOPMO strategy are, therefore, reported in Table 10. The LOPMO model was created using the data for 34 participants (again participants 8, 24, and 28 were excluded, along with participants 17, 18, and 22 as with LOMO-Within). For each testing fold, the data from a single participant and movie, both unseen during training, were used to test the model. All data related to this movie (irrespective of participant) and participant (irrespective of movie) were excluded from training. Therefore, the training set consisted of the data for all other movies and participants.

Again, the benefit of feature selection was supported by the statistically significant differences were found between using all 217 features and the selected subsets for both valence and arousal with LDA, SBS (*p* < 0.005 and *p* < 0.004, respectively), and for arousal with LDA, SFS (*p* < 0.019). Although trending, no statistical significance was found for the SFS features using LDA for valence (*p* = 0.094). For SVM, the features selected from SFS and SBS resulted in significantly different performances than ALL features, except for arousal, SFS ([SFS, SBS]: arousal *p* = [0.387, 0.023], valence *p* = [0.013, 0.010]). CLSTM outperformed other classifiers in valence with an F1-score of 0.639. The arousal results for CLSTM (F1-score: 0.682) were similar to the best performance recorded using LDA. Using the Kruskal–Wallis H-test, the CLSTM results were compared against the CLF, ALL results. Significant differences were found ([LDA, SVM]: arousal *p* = [0.000, 0.000], valence *p* = [0.000, 0.000]).

Table 11 shows the top five features selected for SFS and SBS. When classifying valence using SFS, the top five features obtained 84% of the maximum F1-score for that condition. This is in contrast to the SFS arousal and SBS results, as well as those from the other evaluation frameworks, which all described over 87% of the maximum F1-scores.

**Figure 3:**
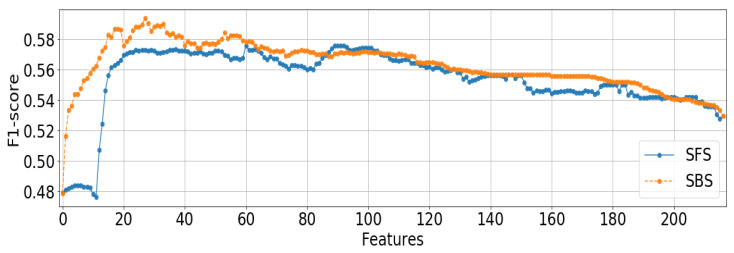
Comparison of SFS and SBS for Valence, LOPMO Validation Using LDA Classification.

Figure 3 demonstrates a comparison of the feature selection results between SFS and SBS for the LOPMO case. Of note is the stark contrast for lower numbers of features until SFS happens to find features whose combinations drastically increase its performance, suggesting the importance of groupings of features in the classification of LOPMO emotion.

### 4.4. Comparative Performance Analysis

In this section, a comparative performance analysis is given between the results obtained in this work and those previously reported in the literature. Most relevant is the original AMIGOS work by [29], in which a set of 105 EEG features were extracted and reduced using a Fisher’s linear discriminant (FLD) approach. Naive Bayes classifiers were used to create the emotion recognition models for valence and arousal and obtained LOPO cross-validation F1-scores for the long movies of 0.557 and 0.571, respectively. These results, along with the best results from the various cross-validation techniques evaluated in this work, are summarized in Table 12. This table shows that, despite testing more challenging cross-validation frameworks, the obtained feature-based results outperform those reported by the original AMIGOS LOPO work.

For comparison, we also include the results from a variety of other works using the unimodal EEG data from AMIGOS in Table 13. Only works that presented results using F1-score are included in this table. Again, all of these results reflect the performance within the LOPO cross-validation scheme. It should be noted, however, that these works are not directly comparable because they either used short movies [24,83,84] or a combination of both short & long [18] for training their system. Moreover, Siddarth et al. [24] used the subject’s self-reported labels in their study thus assuming labels to be static throughout the full movie. In [83,84], emotion levels were split into 2 classes based on the mean of the assessed values. Furthermore, they dropped 7 subjects from their analysis without disclosing the participant-IDs. In [18], Miranda et al. used the median affect value as a threshold to divide the samples into high and low classes. Here, we followed the original AMIGOS [29] approach to divide the high-low valence and arousal classes and used the same number of participants, making the results more comparable. Despite this, the results presented here for the long movies still outperform most previous short movie results (as well as both previous long-movie results).

## 5. Discussion

Mental health challenges are a growing problem globally but have been shown to respond well to early intervention. Detecting consistent or large swings in emotion could be an important indicator as part of a prevention program. Affective computing provides the means to understand and monitor emotion objectively using automated systems and devices. Many emotion recognition techniques have been explored; however, interpretability of results remains limited but crucial for clinical adoption. In this work, we presented an analysis of EEG-based emotion recognition across a variety of conditions and use cases. The results from the various cross-validation metrics showed the benefit of feature selection and that SFS often does not perform as well as SBS. This is likely because there are important relationships between EEG-based features that are only captured by SBS. For both feature selection techniques, the first five features provided around 90% of the maximum information to the classifier. Furthermore, the additional features extracted in this work were frequently chosen by the various models, improving upon previously reported results. Of the extracted features, HFD was chosen most frequently as the first feature. Alarcao’s review of emotion recognition using EEG signals [12] found that the most commonly used features in the literature include PSD (22.2%), and entropy (15.9%), whereas fractal dimensions are only used in 7.9% of works. As documented in [70], although the Fast Fourier transformation (FFT), used for PSD, is beneficial for the analysis of stationary signals, neurophysiological processes are often non-stationary. This work corroborates those of [45,82] that HFD is informative for EEG emotion recognition tasks.

The chosen features also align with reports from neuropsychology, with temporal lobe channels frequently being chosen as a top feature, followed by frontal lobe channels. The temporal lobe is critical for this type of emotion recognition as it plays a role in audio, visual and emotion processing. The frontal lobes play key roles in emotion retrieval, as well as audio processing; therefore, it makes sense that the majority of the chosen channels come from these lobes. However, in contrast, it should be noted that Rayatdoost et al. recently suggested that the emotion information from the temporal and frontal EEG electrodes may also be contaminated by muscle artefacts caused by facial expression [38,85].

The best performing results, shown in Table 12, come from SVM, LDA, and CLSTM classifiers. Although the LOPMO case was the worst performing of all the cross-validation techniques in this work, our results still outperformed the original AMIGOS results for LOPO. The use of LOPMO arguably yields the best reflection of potential real-world performance and these results suggest that there is benefit to be gained by designing specifically for this case. Though both LDA and SVM were implemented for each feature selection technique and affect type, there were no statistically different performances once feature selection had been completed, while the participant-dependent analysis using LOMO-Within did not outperform the independent model of LOMO-Inter, the reasons this could have occurred include the chosen cross-validation technique, the relatively small amount of data, or the imbalance in the labels. The deep-learning CLSTM model, whose hyperparameters were optimized for the LOPO CV scheme, struggled (comparatively) to generalize to the LOMO cross-validation models. This is likely due to the reduced training data in these validation schemes, but may also benefit from additional tuning for these use cases. Nevertheless, the amount of data needed to train any within-subject implementation remains an in impediment to practical usability, further supporting the recommendation to focus future works on the LOPMO CV scheme. Furthermore, the high inter-subject variability, combined with a desire to reduce training burden, motivates the continued exploration of transfer learning algorithms (e.g., domain adaptation) [86,87].

## 6. Conclusions

This work described the design and analysis of EEG-based emotion recognition systems for time-varying emotion. The original AMIGOS work was extended by extracting an additional 112 features and exploring wrapper-style feature selection. Additionally, three lesser-used cross-validation techniques were evaluated to understand the robustness of the models under different combinations of un/known subjects and un/known stimuli. Consensus was found between the four models, suggesting that HFD features and information from the temporal lobe are important in the classification of emotion from EEG. As the techniques became more generalizable, a performance drop was noted, yet this was marginal for arousal. The exception to this trend was LOMO-Within, which likely suffered as a result of the few movies per participant and severe class imbalance. In the future, additional features that focus on the temporal information and other feature explainable (e.g., SHAP values) and classification schemes (e.g., fuzzy classifier) could be explored to improve the system performance. Additionally, other modalities could also be introduced to create a multi-modal system, as multi-modal systems have been shown to improve the emotion recognition performance. Perhaps most importantly, new AMIGOS-like datasets are needed to advance the field, but with more subjects and with more data per subject.

## Figures and Tables

**Table 1:** Details of the movies used to record time-varying emotions EEG data.

Dataset	Movie Details	Duration
AMIGOS [29], EEG-14 Channels, 128 Hz	The Descent. Dir. Neil Marshall. Lionsgate. 2005	23:35
	Back to School Mr. Bean. Dir. John Birkin. Tiger Aspect Productions. 1994.	18:43
	The Dark Knight. Dir. Christopher Nolan. Warner Bross. 2008.	23:30
	Up. Dirs. Pete Docter and Bob Peterson. Walt Disney Pictures and Pixar Animation Studios. 2009	14:06

**Table 2:** Cross-validation Evaluation Frameworks.

	Known Subject	Unknown Subject
Known Stimulus	Subject- and stimulus-dependent (k-fold, LOO)	Subject-independent (LOPO)
Unknown Stimulus	Stimulus-independent (LOMO)	Subject- and Stimulus-independent (LOPMO)

**Table 3:** Cross-validation Train/Test size details. Note: each experiment was repeated several times based on the cross validation scheme.

Validation Scheme		#Users	#Train (Users ∗ Movies)	#Test (Users ∗ Movies)
LOPO		37, 4 movies	36 ∗ 4	1 ∗ 4
LOMO	Inter	37, 4 movies	37 ∗ 3	37 ∗ 1
	Within	Valence-26 Arousal-19	1 ∗ 3	1 ∗ 1
LOPMO		34, 4 movies	33 ∗ 3	1 ∗ 1

**Table 4:** LOPO (Participant-Independent) Results for Each Classifier (CLF) and Feature Selection (FS) Technique. [*, ^†^] denotes a significant difference from the respective [LDA, SVM] ALL results (*p* < 0.05).

CLF, FS	Valence		Arousal	
	*F1*	*#Features*	*F1*	*#Features*
LDA, SFS	0.687	24	0.683	168
LDA, SBS	0.695	77	**0.699**	63
LDA, ALL	0.648	217	0.664	217
SVM, SFS	0.680	72	0.676	75
SVM, SBS	0.706 ^†^	45	0.694	49
SVM, ALL	0.648	217	0.643	217
CLSTM	**0.741** *^,†^	-	0.677	-

**Table 5:** Top Five Features Selected by SFS and SBS for the LOPO Validation Framework. Note: **bold features** were not used in the original AMIGOS set.

		LDA		SVM	
		*Valence*	*Arousal*	*Valence*	*Arousal*
SFS	1	**T7 HFD**	**T7 HFD**	T7 PSDγ	T7 PSDγ
	2	**AF4 HFD**	**AF4 HM**	AF4 PSDγ	**P8 HFD**
	3	**T8 HFD**	T7 PSDα	**P7 HFD**	**AF3 HFD**
	4	**F8 PFD**	T7 PSDβ	FC Asymmγ	T7 PSDβ
	5	**FC6 HM**	**T8 HFD**	F34 Asymmθ	**F4 SpecEnt**
		0.651 (95%)	0.643 (94%)	0.644 (95%)	0.632 (94%)
SBS	1	**T8 HFD**	**T8 HFD**	**T8 HFD**	**T7 HFD**
	2	**AF4 HFD**	**AF4 HFD**	FC5 PSDθ	**F7 svdEnt**
	3	**F7 PFD**	T7 PSDγ	FC5 PSDγ	FC5 PSDα
	4	**FC5 HM**	T7 PSDβ	F7 PSDγ	**F7 HFD**
	5	**O2 HC**	**F8 HM**	**O2 HC**	FC5 PSDβ
		0.633 (91%)	0.620 (89%)	0.639 (91%)	0.629 (91%)

**Table 6:** LOMO-Inter (Participant-Independent) Results for Each Classifier (CLF) and Feature Selection (FS) Technique. No statistically significant differences (*p* > 0.05) were found.

CLF, FS	Valence		Arousal	
	*F1*	*#Features*	*F1*	*#Features*
LDA, SFS	0.636	119	0.675	99
LDA, SBS	0.646	68	**0.684**	63
LDA, ALL	0.604	217	0.647	217
SVM, SFS	0.632	115	0.650	131
SVM, SBS	**0.648**	73	0.657	67
SVM, ALL	0.610	217	0.626	217
CLSTM	0.612	-	0.603	-

**Table 7:** Top Five Features Selected by SFS and SBS for the LOMO-Inter Validation Framework. Note: **bold features** were not used in the original AMIGOS set.

		LDA		SVM	
		*Valence*	*Arousal*	*Valence*	*Arousal*
SFS	1	T7 PSDγ	T8 PSDγ	T7 PSDγ	T8 PSDγ
	2	AF3 PSDβ	**T7 HFD**	AF4 PSDβ	**T7 HFD**
	3	**F8 HFD**	**F7 HM**	**T8 HFD**	**AF4 HC**
	4	**AF4 HC**	**T7 svdEnt**	**AF4 HFD**	**T7 svdEnt**
	5	**T8 HFD**	FC5 PSDβ	**F8 HFD**	P Asymmγ
		0.571 (90%)	0.608 (90%)	0.604 (96%)	0.616 (95%)
SBS	1	**T7 HFD**	**T8 HFD**	**T7 HFD**	**T7 HFD**
	2	**AF4 HFD**	T8 PSDθ	**AF4 HFD**	**F7 svdEnt**
	3	**T8 HFD**	T7 PSDβ	F8 PSDγ	**F7 HFD**
	4	F7 PSDγ	T7 PSDγ	**T8 HFD**	FC5 PSDα
	5	FC5 PSDβ	P8 PSDβ	F8 PSDβ	FC5 PSDβ
		0.578 (90%)	0.598 (87%)	0.607 (94%)	0.603 (92%)

**Table 8:** LOMO-Within (Participant-Dependent) Results for Each Classifier (CLF) and Feature Selection (FS) Technique. [*, ^†^] denotes a significant difference from the corresponding [LDA, SVM] ALL results (*p* < 0.05); ^‡^ denotes a significant difference between the LDA and SVM results (*p* < 0.05).

CLF, FS	Valence		Arousal	
	*F1*	*#Features*	*F1*	*#Features*
LDA, SFS	**0.596** *	20	**0.657** *	20
LDA, SBS	0.592 *	13	0.642 *	20
LDA, ALL	0.445	217	0.470	217
SVM, SFS	0.593 ^†^	53	0.616 ^†^	35
SVM, SBS	0.578 ^†^	30	0.612 ^†^	26
SVM, ALL	0.481	217	0.528 ^‡^	217
CLSTM	0.543 *^†^	-	0.579 *^†^	-

**Table 9:** Top Five Features Selected by SFS and SBS for the LOMO-Within Validation Framework. Note: **bold features** were not used in the original AMIGOS set.

		LDA		SVM	
		*Valence*	*Arousal*	*Valence*	*Arousal*
SFS	1	**T7 HFD**	**T7 HFD**	**T7 HFD**	**T7 PFD**
	2	AF4 PSDγ	FC6 PSDθ	T8 PSDγ	**P8 PFD**
	3	T8 PSDβ	AF3 PSD slowα	**O2 DFA**	**P7 PFD**
	4	**FC6 HFD**	AF3 PSDγ	**AF4 FI**	**F7 PFD**
	5	P Asymmγ	**T7 FI**	**AF4 PFD**	**O1 PFD**
		0.569 (95%)	0.606 (92%)	0.574 (97%)	0.549 (89%)
SBS	1	**T7 HFD**	**T7 HFD**	T8 PSDγ	T8 PSDγ
	2	AF3 PSDγ	**AF3 HFD**	**O2 HC**	T Asymmγ
	3	**T8 HFD**	AF3 PSD slowα	AF3 PSDγ	AF3 PSDθ
	4	T8 PSDθ	F4 PSDβ	T Asymmγ	P7 PSDγ
	5	**AF4 FI**	**F7 HFD**	F8 PSDγ	**P7 HC**
		0.560 (95%)	0.616 (96%)	0.539 (93%)	0.562 (92%)

**Table 10:** LOPMO (Participant- and Movie-Independent) Results for Each Classifier (CLF) and Feature Selection (FS) Technique. [*, ^†^] denotes a significant difference from the respective [LDA, SVM] ALL results (*p* < 0.05); ^‡^ denotes a significant difference between the LDA and SVM results (*p* < 0.05).

CLF, FS	Valence		Arousal	
	*F1*	*#Features*	*F1*	*#Features*
LDA, SFS	0.575	61	0.670 *^‡^	102
LDA, SBS	0.594 *	28	**0.686** *^‡^	72
LDA, ALL	0.521	217	0.605	217
SVM, SFS	0.586 ^†^	32	0.593	123
SVM, SBS	0.578 ^†^	49	0.624 ^†^	27
SVM, ALL	0.511	217	0.574	217
CLSTM	**0.639** *^†^	-	0.682 *^†^	-

**Table 11:** Top Five Features Selected by SFS and SBS for the LOPMO Validation Framework. Note: **bold features** were not used in the original AMIGOS set.

		LDA		SVM	
		*Valence*	*Arousal*	*Valence*	*Arousal*
SFS	1	**FC6 HM**	**T8 HFD**	P8 PSDγ	**T7 HFD**
	2	F8 PSDα	T8 PSD slowα	**T7 HFD**	F7 PSDθ
	3	**O1 DFA**	**AF3 svdEnt**	**AF3 HFD**	**AF3 svdEnt**
	4	AF Asymmθ	**T7 HFD**	**T8 HFD**	O2 PSDθ
	5	P Asymmθ	**F4 HFD**	T8 PSDθ	F8 PSDα
		0.484 (84%)	0.630 (94%)	0.571 (97%)	0.576 (97%)
SBS	1	**AF4 HFD**	**T8 HFD**	**T7 HFD**	**T8 HFD**
	2	**T7 HFD**	T8 PSD slowα	**AF3 HFD**	T8 PSDθ
	3	**T8 HFD**	T7 PSDβ	**T8 HFD**	**T7 HFD**
	4	AF3 PSDγ	T7 PSDγ	**T7 svdEnt**	**F4 HFD**
	5	P8 PSDγ	AF3 PSDα	T8 PSDα	FC5 PSDβ
		0.544 (92%)	0.626 (91%)	0.561 (97%)	0.587 (94%)

**Table 12:** Best Feature-Based and Corresponding CLSTM Results for each Cross-validation Techniques.

	Valence					Arousal				
	*Precision*	*Recall*	*F1*	*CLF*	*FS*	*Precision*	*Recall*	*F1*	*CLF*	*FS*
AMIGOS [29]			0.557	NB	FLD			0.571	NB	FLD
LOPO	0.729	0.722	0.706	SVM	SBS	0.794	0.691	0.699	LDA	SBS
	0.779	0.738	0.741	CLSTM	-	0.743	0.695	0.677	CLSTM	-
LOMO- Inter	0.643	0.731	0.648	SVM	SBS	0.716	0.682	0.684	LDA	SBS
	0.649	0.649	0.612	CLSTM	-	0.635	0.63	0.603	CLSTM	-
LOMO- Within	0.635	0.638	0.596	LDA	SFS	0.700	0.692	0.657	LDA	SFS
	0.612	0.611	0.543	CLSTM	-	0.636	0.666	0.579	CLSTM	-
LOPMO	0.666	0.655	0.594	LDA	SBS	0.738	0.716	0.686	LDA	SBS
	0.711	0.710	0.639	CLSTM	-	0.747	0.735	0.682	CLSTM	-

**Table 13:** Previous F1-score LOPO Results Using the Unimodal EEG Data From AMIGOS.

Source	CLF	Feats	Length	F1-Score *Valence Arousal*	
AMIGOS 2018 [29]	NB	PSD & Asymm	Long	0.557	0.571
			Short	0.576	0.592
Siddharth et al., 2019 [24]	ELM	PSD, Deep & Entropy	Short	0.800	0.740
Miranda-Correa and Patras 2018 [18]	CNN	PSD & EEG-sequence	Short & Long	0.580	0.570
	RNN			0.570	0.590
	Fusion			0.590	0.610
Wang et al., 2018 [84]	XGB	PSD & Asymm	Short	0.577	0.604
	SVM			0.556	0.557
Tung et al., 2019 [83]	XGB (1)	Enrtopy-domain	Short	0.575	0.568
	XGB (2)			0.753	0.568

## Data Availability

Not applicable.
